# Down-regulation of *Fra a 1.02* in strawberry fruits causes transcriptomic and metabolic changes compatible with an altered defense response

**DOI:** 10.1038/s41438-021-00492-4

**Published:** 2021-03-10

**Authors:** Begoña Orozco-Navarrete, Jina Song, Ana Casañal, Rosangela Sozzani, Victor Flors, José F. Sánchez-Sevilla, Johanna Trinkl, Thomas Hoffmann, Catharina Merchante, Wilfried Schwab, Victoriano Valpuesta

**Affiliations:** 1 Laboratorio de Bioquímica y Biotecnología Vegetal, Instituto de Hortofruticultura Subtropical y Mediterránea (IHSM), Universidad de Málaga-Consejo Superior de Investigaciones Científicas, Departamento de Biología Molecular y Bioquímica, Facultad de Ciencias, UMA, Málaga, Spain; 2 Department of Plant and Microbial Biology, North Carolina State University, Raleigh, NC USA; 3 Metabolic Integration and Cell Signalling Group, Plant Physiology Section, Department of Ciencias Agrarias y del Medio Natural, Universitat Jaume I, Castelló, Spain; 4 Departamento de Genómica y Biotecnología, IFAPA, Málaga, Spain; 5 Biotechnology of Natural Products, Technische Universität München, Liesel-Beckmann-Str. 1, 85354 Freising, Germany

**Keywords:** Biotic, Plant molecular biology

## Abstract

The strawberry Fra a 1 proteins belong to the class 10 Pathogenesis-Related (PR-10) superfamily. In strawberry, a large number of members have been identified, but only a limited number is expressed in the fruits. In this organ, Fra a 1.01 and Fra a 1.02 are the most abundant Fra proteins in the green and red fruits, respectively, however, their function remains unknown. To know the function of Fra a 1.02 we have generated transgenic lines that silence this gene, and performed metabolomics, RNA-Seq, and hormonal assays. Previous studies associated Fra a 1.02 to strawberry fruit color, but the analysis of anthocyanins in the ripe fruits showed no diminution in their content in the silenced lines. Gene ontology (GO) analysis of the genes differentially expressed indicated that oxidation/reduction was the most represented biological process. Redox state was not apparently altered since no changes were found in ascorbic acid and glutathione (GSH) reduced/oxidized ratio, but GSH content was reduced in the silenced fruits. In addition, a number of glutathione-S-transferases (GST) were down-regulated as result of Fra a 1.02-silencing. Another highly represented GO category was transport which included a number of ABC and MATE transporters. Among the regulatory genes differentially expressed WRKY33.1 and WRKY33.2 were down-regulated, which had previously been assigned a role in strawberry plant defense. A reduced expression of the VQ23 gene and a diminished content of the hormones JA, SA, and IAA were also found. These data might indicate that Fra a 1.02 participates in the defense against pathogens in the ripe strawberry fruits.

## Introduction

The family of Fra a 1 proteins in strawberry (*Fragaria* spp) belong to the superfamily of Pathogenesis-Related class 10 (PR-10) proteins. PR-10 proteins are constitutively expressed in the cytoplasm of different plant tissues and organs and are frequently up-regulated after biotic and abiotic stress^1^. Different molecular roles have been proposed for some members of the PR-10 family, mostly based on in vitro studies. Some examples include their involvement in enzymatic processes, biosynthesis of secondary metabolites, and binding, storage and transport of phytohormones and other hydrophobic ligands^1^. However, a unique biological function has not been assigned to these proteins yet.

A large number of PR-10 genes have been identified in different species with expression patterns that differ between normal growth and stress conditions^2,3^. In strawberry, 39 *Fra a 1* genes have been identified to date^4^. Transcriptome analysis of the ripening fruit, achenes and receptacle separately, and vegetative tissues showed different expression patterns for each *Fra a 1* gene^5^. In these tissues, *Fra a 1.01* to *Fra a 1.08*^6,7^ are highly expressed. In the achenes, *Fra a 1.01* showed the highest expression level followed by *Fra a 1.03*. In the receptacle, *Fra a 1.01* and *Fra a 1.02* were highly abundant, with *Fra a 1.01* peaking at the green stage and *Fra a 1.02* at the red stage^5^. Transcripts of *Fra a 1* genes are present in most strawberry organs, with expression levels depending on the member of the family^8^. Within the species, there is also a wide variability in the content of Fra a 1 proteins in ripe fruits^9^.

The specific functions of Fra a 1 proteins are largely unknown, but they could play a role in the response to biotic and abiotic stresses, as members of the PR10 superfamily^2^. Fra a 1 have been associated with color development, since proteomic studies showed a lower content of these protein in white-fruited strawberry varieties compared to the red ones^10^. However, a new study among different strawberry genotypes has not been able to correlate color of the ripe fruits with the Fra a 1 content^9^. Recently, it has been reported that some of the Fra a 1 proteins, Fra a 1.04 to Fra a 1.08, present RNase activity depending on their phosphorylation state^3^. The RNase activity of these proteins could be related to a possible role in the plant defense against different pathogens.

The PR10 family is well characterized at the structural level^1^. These proteins share a common fold with the START domain, which is characterized by a seven-stranded beta sheet that encloses a central hydrophobic cavity. This cavity also presents some polar residues that facilitate the binding of small ligands^1^. Several crystal structures of Fra a 1.01E, Fra a 1.02 and Fra a 1.03, the last one bound to catechin, have been resolved^11,12^. These structures show high similarity, and they all exhibit the characteristic START fold. Previous studies showed that these three Fra a 1 proteins bind specific metabolites of the flavonoid pathway with affinities in the low μM range^11^. In addition, a flavonoid glycoside has been identified as natural ligand of Mal d 1, a homologous protein to Fra a 1 from apple^13^. The functional relevance of these interactions remains unclear, but the specificity of the interactions and the subtle conformational change associated to the natural ligand binding^11,12^ suggest that Fra a 1 proteins could play a regulatory role in intracellular signaling.

To advance our understanding of the function of Fra a 1 in the ripe strawberry fruit, we have produced transgenic strawberry plants that silence, by RNAi, *Fra a 1.02*. Other members of the gene family were also silenced at different degree. Transcriptomic, metabolomic and hormone level analysis of the silenced ripe fruits support a role of Fra a 1.02, the member of the family with highest expression in the red fruits^5,8^, in the defense of the plant against pathogens.

## Results

### **S**ilencing of *Fra a 1.02* in strawberry fruits has no effect on fruit color development

**Fig. 1:**
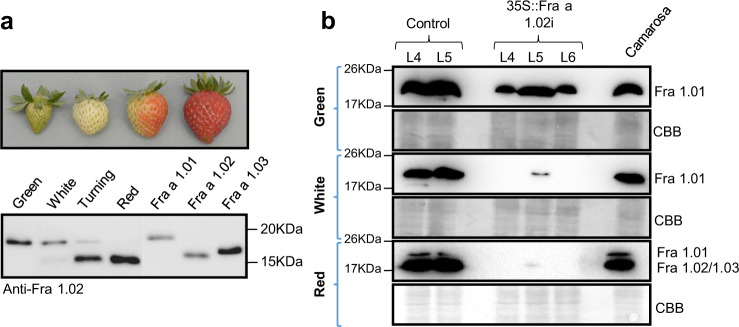
Analysis of Fra a 1 proteins in strawberry fruits along ripening and in transgenic lines. **a** Photographs of fruits representative of green, white, turning and red stages of fruit development, and western blot analysis of extracts from strawberry receptacles at these stages, using anti-Fra a 1.02 antibodies. FaFra a 1.01, FaFra a 1.02, and FaFra a 1.03 recombinant proteins, with a 6X His tag, purified from *E. coli* were used as controls. Equal amounts of total protein extracts from strawberry were loaded. **b** Western blot of fruit extracts from transgenic lines L4, L5, L6, at three developmental stages, using anti-Fra a 1.02 antibodies. Controls correspond to plants transformed with the empty vector. A positive control of ripe fruit extract of the cv. Camarosa is included. Membranes stained with Coomassie Brilliant Blue (CBB) are also shown for each stage sample.

The analysis of Fra a 1 proteins in the receptacle of strawberry fruits (cv. Camarosa) at different developmental stages (Fig. 1a) confirmed the highest expression of *Fra a 1.02* in the ripe fruits^5,8^. Antibodies were generated against Fra a 1.02, but they also recognize other Fra a 1 proteins due to the high similarity of their protein sequences. Thus, the three Fra a 1 proteins, which were most highly expressed along fruit development and ripening^8^ were detected by this antibody. Differences in size allowed to distinguish Fra a 1.01 from Fra a 1.02 and Fra a 1.03. A silencing construct was designed against the *Fra a 1.02* gene using the 124 bp fragment of the coding region of *Fra 1.02* that showed the highest specificity for the gene (Figure S1). Since other *Fra* *a 1* genes present high homology to *Fra a 1.02*, and are also expressed in the strawberry fruits, it is expected some of them will be co-silenced together with our target gene^5,6^ Strawberry plants were transformed with the *Fra a 1.02 RNAi* construct (*35* *S::Fra a 1.02i*) and with an empty vector (*pBINPLUS)* as a control. For the analysis, three independent transgenic lines transformed with the *Fra a 1.02 RNAi* construct, and two control lines were selected.

Western blot analysis of proteins isolated from the transformed fruits, at different stages, including protein extracts of fruits of untransformed strawberry plants (cv. Camarosa), was performed (Fig. 1b). At the green stage, the Fra a 1.01 protein is highly abundant (Fig. 1a) but we observed a diminished content of this protein in the 35S::Fra a 1.02i fruits. At the white stage, Fra a 1.01 was only detectable in one of the 35S::Fra a 1.02i fruits as a weak band (Fig. 1b, 35S::Fra a 1.02i-L5). At the red stage, when expression of Fra a 1.02 is highest in the control plants, only a very faint band is detected in the silenced L5 line (Fig. 1b, 35S::Fra a 1.02i-L5). The efficient *Fra a 1* silencing, especially of *Fra a 1.02* in ripe fruits, prompted us to analyze these fruits to investigate the role of Fra a 1.02 in the ripe strawberry fruits.

**Fig. 2:**
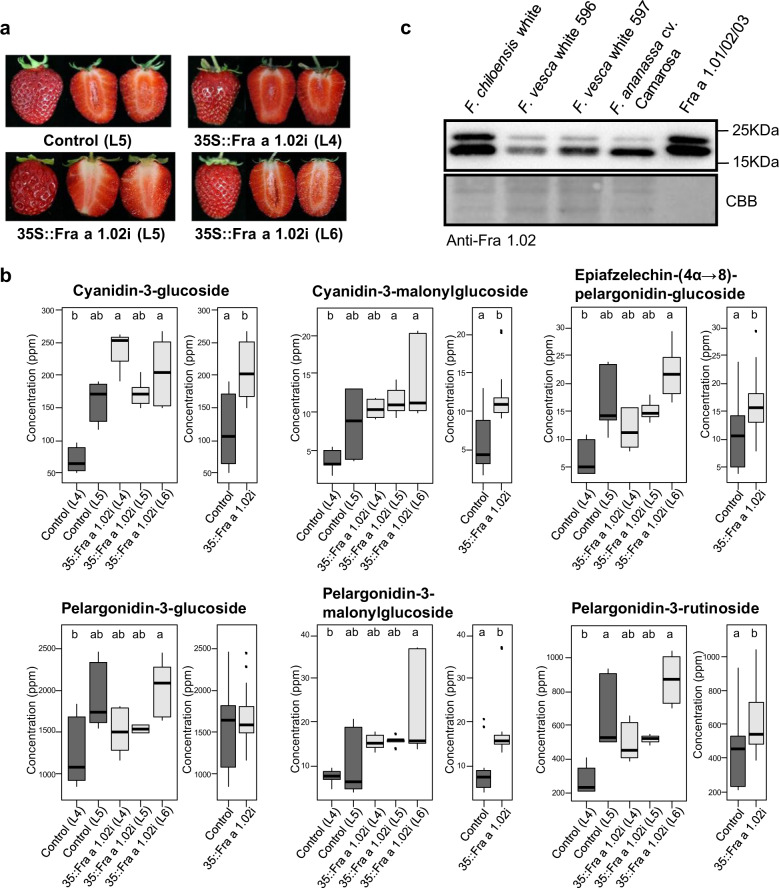
Effect of Fra a 1.02 silencing in the content of anthocyanins of the fruit. **a** Ripe fruits of the control (L5) and the transgenic lines (L4, L5, L6) silenced with the *Fra a 1.02 RNAi* construct. **b** Concentration of selected anthocyanins in the fruits of independent transgenic lines, and the average of the two controls and the three *Fra a 1.02-*silenced lines. Data for each line are the average of three biological repetitions, each with two technical repetitions. Concentrations are expressed as equivalents of g/kg (ppm) 4-methylumbelliferyl glucoside (dry weight). For significant differences (different letters) a Kruskal–Wallis test was performed followed by a Dunn-Bonferroni-Test using a *p*-value of ≤0.05. **c** Western blot of ripe fruit extracts from a white variety of *Fragaria chiloensis*, two white accessions of *Fragaria vesca* and the cv. Camarosa of *Fragaria x ananassa*. Purified Fra a 1 proteins produced by heterologous expression in *E. coli* is included. Membranes stained with Coomassie Brilliant Blue (CBB) are also shown for each stage sample.

Interestingly, during the ripening process there was no phenotypic difference between the controls and the silenced lines. Thus, size and color of the ripe fruits of the transgenics and controls were not different (Fig. 2a). Since anthocyanins are the main compounds responsible for strawberry fruit color, their contents were analyzed, jointly with other flavonoids and metabolites present in the strawberry fruit (Table S1), in the control and the silenced fruits at the ripe stage. The results showed that there were no clear differences between the two sets of lines, but the comparison of the mean values showed that the fruit content of some cyanidin and pelargonidin derivatives is even slightly higher in the silenced lines (Fig. 2b). This was relevant since the primary assignment of a function to the Fra a 1 proteins in strawberry fruits was associated with color development, based on their low level/absence in the white varieties of this species^10^. To further investigate the content of Fra a 1 protein in different strawberry varieties, we performed western blots in ripe fruits of white-fruited *F. chiloensis* and two accessions of *F. vesca* (Fig. 2c). We used ripe fruits of *F. ananassa* (cv. Camarosa) as control as well as a mix of equivalent amounts of Fra a 1.01, Fra a 1.02, and Fra a 1.03 produced in *E. coli*^11^. Fra a 1 proteins were detected in all the samples, at different levels, even in the white varieties (Fig. 2c).

### RNAseq analysis of silenced fruits identify redox processes and membrane components as major categories of differentially expressed genes

RNAseq was performed on RNA isolated from the same set of fruits used for the metabolic analysis (*i.e*. the three independent silenced lines and the two controls) to analyze the transcriptome of ripe fruits (Table S2). First, the expression of the *Fra a 1* genes^3^ was examined. *Fra a 1.02* expression was down-regulated by more than 92 percent, as were *Fra a 1.04*, *Fra a 1.05*, and *Fra a 1.06* (Figure S2). The other set of *Fra a 1* genes, *Fra a 1.01*, *Fra a 1.03*, and *Fra a 1.07* were down-regulated by more than 50 percent, and *Fra a 1.08* by 22 percent. The dendrogram obtained from the alignment of the DNA sequences (Figure S2) showed the observed degree of silencing correlated well with the sequence homology between *Fra a 1* genes. This result points to a silencing effect of the hairpin generated from the RNAi construct, and the similarity among the DNA sequences (Figure S1).

**Fig. 3:**
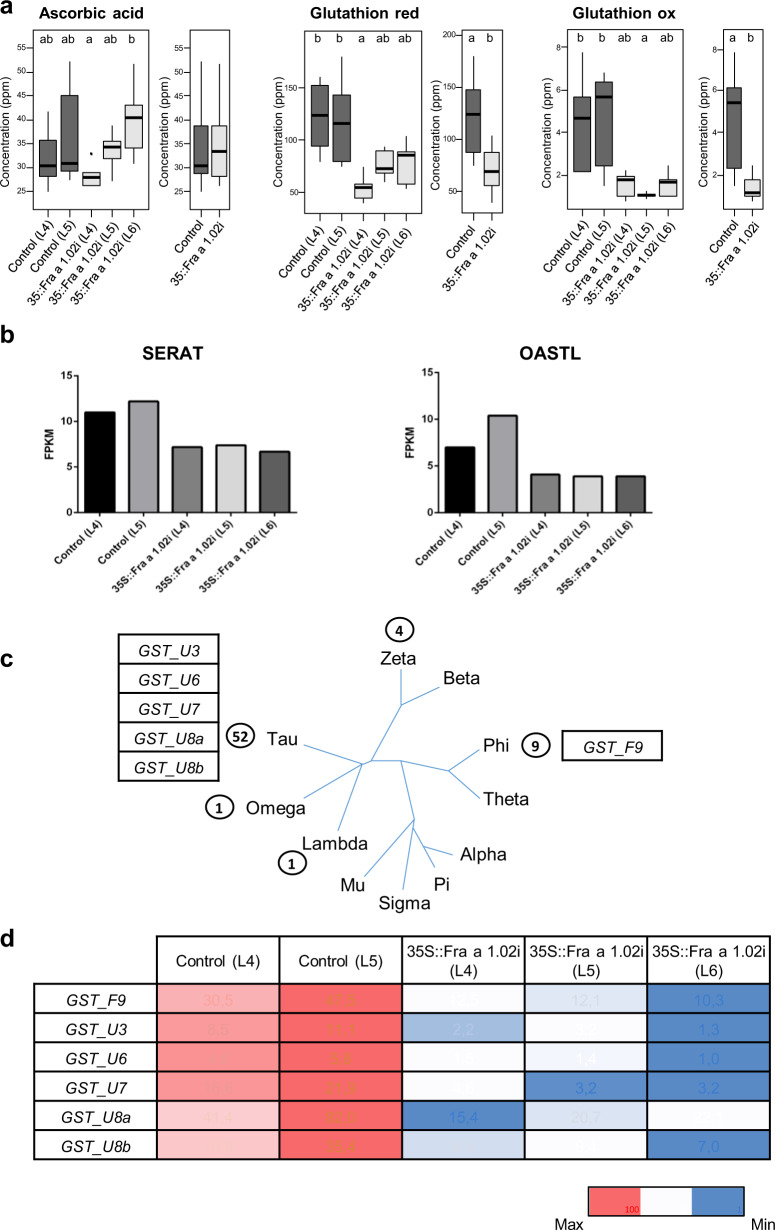
Redox compounds and expression of glutathione-related genes in the transgenic lines. **a** Concentration of ascorbic acid and reduced and oxidized glutathione in the fruits of independent transgenic lines, and the average of the two controls and the three *Fra a 1.02*-silenced lines. Data for each line are the average of three biological repetitions, each with two technical repetitions. Concentrations are expressed as equivalents of g/kg (ppm) 4-methylumbelliferyl glucoside (dry weight). For significant differences (different letters) a Kruskal–Wallis test was performed followed by a Dunn-Bonferroni-Test using a *p*-value of ≤0.05. **b** Expression values of genes of cysteine biosynthesis differentially expressed in the three silenced lines compared to the two controls. Values correspond to fragments per kilo base per million mapped reads (FPKM). **c** Insertion of the *GSTs* from *Fragaria vesca* in the different families of GST of the phylogenetic tree generated from Dixon et al.^17^ in Arabidopsis and some model organisms. Circled numbers indicate the *F. vesca* genes in each class. **d** HeatMap representing the expression values of the GSTs differentially expressed in the control and the Fra a 1.02-silended lines. Red and blue color represent the maximum and minimum values, respectively, for each gene. Analysis of differentially expressed genes (**b**–**d**) was performed with Cufflinks, establishing a *p*-value < 0.002 with the Benjamini–Hochberg procedure for multiple comparisons. Values correspond to fragments per kilo base per million mapped reads (FPKM).

Two-hundred and five differentially expressed genes (DEG) were identified in the three silenced lines compared to the two controls (Table S3). Analysis of the GO terms of the DEGs showed that oxidation-reduction was by far the most represented category among the Biological Processes (Table S4, Figure S3). In terms of the Cellular Component the membrane/integral component of membrane was the most strongly represented of the 205 DEGs with 78 percent of the assigned GO terms. The high number of DEGs assigned to the oxidation-reduction process prompted us to measure the content of the two main compounds responsible for the maintenance of the redox status in plants, i.e. L-ascorbic acid and glutathione (GSH). While the content of L-ascorbic acid was not different in the fruits of the control and the silenced lines, the concentration of reduced GSH was diminished in the *Fra a 1.02*-silenced fruits (Fig. 3a). However, the oxidized GSH also decreased in the silenced fruits and, consequently, the redox potential of GSH was not altered (Fig. 3a). Overall, the total GSH content in the transgenic fruits thus decreased compared to the controls.

GSH (γ-Glu-Cys-Gly) is synthesized in plants from the constituent amino acids in two steps, catalyzed by the γ-glutamylcysteine synthetase and the glutathione synthetase^14^. Their corresponding genes were not differentially expressed in the silenced receptacle in comparison with the controls (Table S2) and the concentrations of the precursor amino acids Glu and Gly were not altered in the transgenics (Table S5). However, two genes encoding enzymes of critical steps in the synthesis of Cys, the serine acetyltransferase (SERAT) and the O-acetylserine (thiol) lyase (OASTL) were down-regulated in the silenced fruits (Fig. 3b). Changes in the expression in these genes alter GSH levels in Arabidopsis^15,16^, and could be the cause of the decreased GSH levels in the silencing lines.

Glutathione, in addition to its role in redox signaling, plays important roles in defense and detoxification^14^. In these processes, GSH is conjugated to compounds for detoxification or transport. The reaction is catalyzed the glutathione-S-transferase (GST) enzymes^17^. In the *F. vesca* genome 67 *GST* genes have been identified^7^. The dendrogram generated from their amino acid alignment (Fig. 3c) showed that GSTs from five classes are represented in *F. vesca*^18^. Six GSTs were significantly down-regulated in the *Fra a 1.02*-silenced fruits (Fig. 3d).

**Fig. 4:**
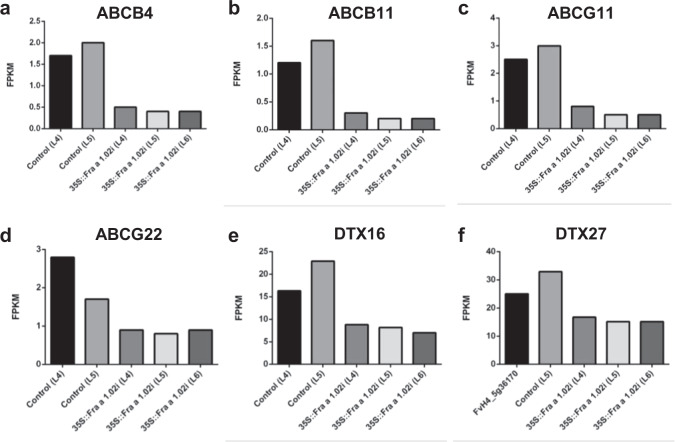
Expression values of transport genes in transgenic fruits. ABC (**a**–**d**) and MATE (**e**, **f**) transporter genes differentially expressed in the three silenced lines compared to the two controls. Analysis of differentially expressed genes was performed with Cufflinks, establishing a *p*-value<0.002 with the Benjamini–Hochberg procedure for multiple comparisons. Values correspond to fragments per kilo base per million mapped reads (FPKM).

Membrane and integral components of membrane represent 68 percent of GO terms for cell components, and transmembrane transport is among the most represented GO terms of the biological processes (Table S4). We found that transcript levels of four members of the ABC family of transporters were down-regulated in the silenced fruits compared to the controls (Fig. 4a-d). In addition, two members of the Multidrug and Toxic Compound Extrusion (MATE) transporter, also known as Detoxification Efflux Carrier (DTX), were down-regulated in the fruits of the *Fra a 1.02*-silenced lines (Fig. 4e,f). In plants, these proteins are involved in the transport of secondary metabolites and in the defense response, among other functions^19^.

### Regulatory *WRKY* genes are down-regulated in ripe fruits as result of *Fra a 1.02* silencing

**Fig. 5:**
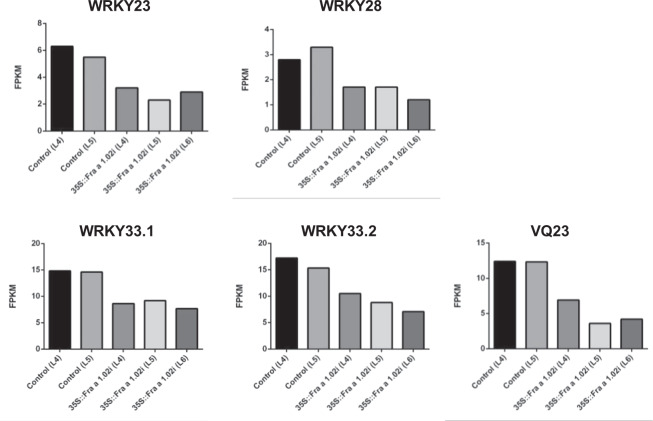
Expression or regulatory defense genes in transgenic fruits. Expression values in fruits of *WRKY* and *VQ* genes differentially expressed in the three silenced lines compared to the two controls. Analysis of differentially expressed genes was performed with Cufflinks, establishing a *p*-value <0.002 with the Benjamini-Hochberg procedure for multiple comparisons. Values correspond to fragments per kilo base per million mapped reads (FPKM).

Regulation of transcription is another GO category highly represented in the DEGs (Fig. S3). Among these regulatory genes, we found four *WRKY* genes, all of them down-regulated in the Fra-silenced fruits (Fig. 5). The *WRKY* genes were named by their closest homolog in Arabidopsis. In this species, *WRKY23* seems to be involved in the auxin transpor^20^, and *WRKY28* and *WRKY33* are probably associated to the defense response^21,22^. In Arabidopsis, two VQ proteins act as co-activator of WRKY33 in its plant defense response^23^. The corresponding strawberry gene, *VQ23*, is also down-regulated in the *Fra a 1.02*-silenced fruits (Fig. 5).

In strawberry, the infection by *Colletotrichum acutatum* caused an enhanced expression of *WRKY33.1* and *WRKY33.2*, as well as higher concentration of both JA and SA^24^. Noteworthy, the expression of the strawberry *VQ23* gene was also up-regulated in strawberry fruits after *Colletotrichum* infection as well as after external treatment with MeJA and SA^24^. Therefore, the expression of genes involved in the biosynthesis and signaling of these hormones was analyzed in the *Fra a 1.02*-silenced fruits.

**Fig. 6:**
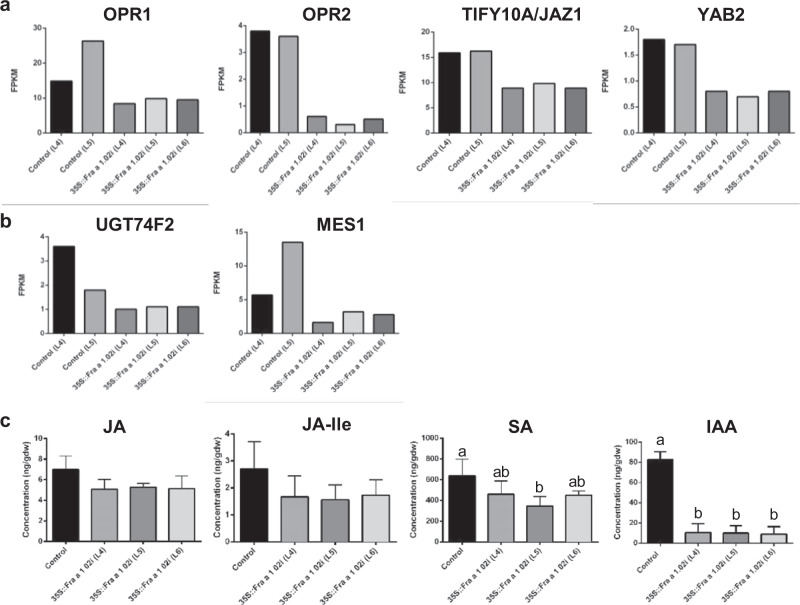
Hormones synthesis and signaling in the transgenic fruits. **a** Expression values of genes of the jasmonic acid (JA) biosynthesis and signaling pathway and **b** of the salicylic acid (SA) biosynthesis differentially expressed in the three silenced lines compared to the two controls. Analysis of differentially expressed genes was performed with Cufflinks, establishing a *p*-value < 0.002 with the Benjamini-Hochberg procedure for multiple comparisons. Values correspond to fragments per kilo base per million mapped reads (FPKM). **c** Hormones content in the receptacle of ripe fruits, expressed in ng per g of dry weight. Same letters include values that are not statistically different by the Tukey test for multiple comparisons. Absence of letters means not statistically significant among lines.

JA biosynthesis starts in the chloroplast by oxidation of linolenic acid to (13S)-hydroperoxyoctadecatrienoic acid, which is converted into cis-(+)-12-oxophytodienoic acid (OPDA) by the activity of two pathway-specific enzymes^25^. The OPDA is reduced by a 12-oxophytodienoate reductase (OPR) enzyme. In the *Fra a 1.02*-silenced fruits two OPR genes, *OPR1* and *OPR2*, are down-regulated (Fig. 6a). In JA signaling the JAZ proteins play a key role, as repressors of the expression of a number of TFs^26^. We found that the expression of *JAZ1*, also named *TIFY10a*, was significantly decreased in the silenced fruits (Fig. 6a). Similarly, a member of the *YABBY* family of TFs (*YAB2*) that have been reported to be targets of JA repression was down-regulated (Fig. 6a). Regarding SA, no changes in expression were found among the key genes involved in its biosynthesis^27^ (Table S2), but two genes involved in its modification were down-regulated (Fig. 6b). In Arabidopsis, the *UGT74F2* gene encodes a glycosyl transferase that catalyzes the glucosylation of SA and the enzyme encoded by *MES1* gene produces a methyl esterase acting on the methyl ester of SA (MeJA)^27^. Interestingly, the Arabidopsis MES1 also hydrolyzes the methyl ester of indole-3-acetic acid (MeIAA)^28^.

The previous results pointed to a decreased synthesis and signaling of JA in the *Fra a 1.02*-silenced fruits. Therefore, the content of JA was measured in the receptacle of the control and the silenced fruits. We restricted the analysis to the receptacle of the ripe fruits, since in this organ and this stage *Fra a 1.02* is by far the most highly expressed member of the family among all the *Fra a 1* genes (Fig. S4). We found that the contents of JA and JA-Ile, the active form of JA, were decreased in the three silenced lines compared to the control (Fig. 6c). Similarly, the concentrations of SA and IAA were decreased in the silenced lines compared to the control (Fig. 6c). In addition, the hormone auxin has been proposed to be involved in the plant defense response against different types of pathogens^29^.

## Discussion

We have transformed strawberry plants with a RNAi construct to silence *Fra a 1.02* and analyze the function of Fra a 1 proteins in strawberry fruit. We should emphasize that the silencing approach has been effective, being an adequate alternative to gene editing, which might be difficult to pursue in this octoploid species, *Fragaria x ananassa*, whose assembled genome is still uncomplete. The analysis of the transcript levels of Fra a 1 proteins in the different transgenic lines showed that the effectiveness of silencing was highest at the red stage of fruit development, where no Fra a 1 proteins were detected (Fig. 1). The transcriptional analysis of the fruits at the red stage showed that silencing of *Fra a 1.02* exceeded 92%. Other *Fra a 1* genes were also down-regulated in the transgenic fruits, and the degree of silencing correlated to their DNA sequence similarity (Fig. S2). Since *Fra a 1.02* is by far the most expressed gene of the *Fra a 1* family in the ripe strawberry fruit^5^, it can be assumed that the molecular effects of silencing in the red fruits reported here are mostly associated with Fra a 1.02.

### Fra a 1.02 influences the cell transport system

Anthocyanins are flavonoid compounds present in the ripe colored fruits of *Fragaria* species. Initially, a role of Fra a 1.02 in the color development in strawberry fruit was proposed, based on the absence of this protein in white species of the *Fragaria* genus^10^, but this correlation was not further confirmed^30,31^. We observed that silencing *Fra a 1.02* in ripe strawberry fruits by more than 92% did not cause a diminution on the content of anthocyanins, but a minor increase was observed when the mean values for control and silencing lines were compared. Anthocyanins are synthesized in the cytosol and their final destination is the vacuole, but the mechanisms for tagging and transport into this organelle have not been fully characterized. At present, two main models have been proposed for the vacuolar sequestration of anthocyanins: a microautophagy-like mechanism and a transporter mediated system where ABC and MATE-type transporters are involved^32,33^. In addition, the involvement of certain GSTs in the transport to the vacuole has been reported^34^. Our analysis of the *Fra a 1.02-*silenced fruits showed that a number of genes encoding for GSTs, ABC, and MATE transporters are down-regulated.

Glutathione transferases (GSTs) are enzymes that catalyze the conjugation of GSH to a variety of molecules, but are also involved in the transport of a number of hydrophobic molecules through membranes, including hormones and, tentatively, anthocyanins^17,18^. Of the six GSTs, which were down-regulated in the *Fra a 1.02*-silenced fruits one belongs to the *Phi* (GSTF) class and five to *Tau* (GSTU) class. A mutant of a member of a GST subfamily (*RAP*) produced white fruits of the woodland strawberry (*Fragaria vesca*)^35^. Transient down-regulation of *RAP* in cultivated strawberry reduced fruit coloration. In this study, we found that a paralog of the *F. vesca RAP* (*RAP_L1*), *GSTF9*, was down-regulated in the silenced fruits, although no difference in fruit coloring was observed in the silenced lines. The expression of *GSTF9* in strawberry is relatively high in root and achene, but low in receptacle^5^. This can explain that a reduction in the expression of this gene in the transgenic lines has no consequence for the content of anthocyanins in the whole fruit.

ABC transporters are membrane proteins that facilitate the exchange of compounds across different membranes, in most cases against electrochemical gradients, thus requiring the hydrolysis of ATP. Anthocyanins have been identified as the transported compounds^32^, Many ABC transporter genes are expressed in the strawberry fruit^5^, but only two members of the subclass B, *ABCB4* and *ABCB11*, and two members of the G subclass, ABCG11 and ABCG22 were down-regulated in the transgenic fruits. The orthologs of these four genes in Arabidopsis have not yet been shown to be involved in transporting anthocyanins. Thus, none of the ABC genes differentially expressed in the *Fra a 1.02* silenced fruits are apparently related to anthocyanin transport to the vacuole.

In the transport-mediated model, some studies support the involvement of multidrug and toxic extrusion (MATE) proteins in the transport of anthocyanins to the vacuole^36^. This large family of cation antiporters is represented by 58 members in Arabidopsis^19^ and 53 in the diploid *Fragaria vesca*^7^. In cultivated strawberry, transient silencing in fruits of a member of the family, *FaTT12_1* (*DTX41*), reduced the content of the proanthocyanidins but not the anthocyanins in the fruits^37^. However, we found that only *DTX16* and *DTX27* were down-regulated in the *Fra a 1.02*-silenced fruits. Although the substrates for some of these transporters are known^19,36^, there is no information on the substrates of DTX16 and DTX27. Some members have been involved in hormone signaling and defense response^38,39^. Thus, the changes observed in the MATE transporters must be considered within the general effects observed in the *Fra a 1.02-*silenced fruits, i.e., there are no significant changes neither in the flavonoids content nor in the redox state of the silenced fruits.

### Fra a 1.02 might be involved in the plant defense response

The protein structure of Fra a 1.01, Fra a 1.02, and Fra a 1.03 have been resolved^11,12^ and natural flavonoids have been identified as potential ligands of these three proteins^11^. Ligand binding is followed by a conformational change of the proteins, which points to a role of the Fra a 1 proteins in the initiation of a signaling pathway in response to a stimulus. The transcriptional analysis of ripe fruits in which Fra a 1 proteins were down-regulated in three silenced lines showed that 205 genes were differentially expressed. Among them, GO categories such as defense, response to biotic stress, and regulation of transcription were among the most strongly represented. The last category included four *WRKY* genes. WRKY TFs play significant roles in regulating plant response to biotic and abiotic stresses, although they also control various developmental and physiological processes^40^. In *F. vesca*, the expression of a gene homologous to *WRKY33* (*FvWRKY42*) was induced in the seedlings after the infection by powdery mildew^41^. In strawberry (*F. x ananassa*) plants, the infection of the crown and the petiole with *Colletothricum acutatum* led to an enhanced expression of *WRKY33.1* and *WRKY33.2*^24^. Interestingly, the *Fra a 1.02* gene was up-regulated in the tissues infected by *Colletothricum*^24^. In parallel, an increase in the JA and SA content of the aerial tissues of the infected plantlets was detected^24^. We found that the content of these two hormones was lower in the three *Fra a 1.02*-silenced fruits. Although this decrease was not significant, the reduced expression of gene involved in JA and SA biosynthesis and metabolism, such as *OPR1*, *OPR2*, *UGT74F2*, and *MES1*^25,27^ was in agreement with an apparent reduction of the hormone contents. In addition, the down-regulation of JA signaling genes such as *JAZ1*, *GL3*, and *YAB2*^26^ further supports the involvement of *Fra a 1.02* in the JA-dependent defense response in ripe strawberry fruits. The crosstalk between these two hormones, JA and SA, in the defense response in the plants, albeit complex, is known^42^, particularly in the strawberry response to *Colletotrichum* infection^24,43^.

It has been reported that auxin is a negative factor for plant defense since elevated auxin signaling correlates with increased susceptibility to biotrophic pathogens^44^. However, we found a significant lower content of auxin in the silenced fruits, what appears to go against a weakened defense response of these fruits. An alternative explanation is the occurrence of a number of defense-related compounds generated from Trp and auxin^29^, whose content would be diminished in a low auxin context. Some indole-derivative phytoalexins and signaling compounds are key elements in the resistance against necrotrophic fungi^45^. It is necessary a more detailed study to know whether the total pool of indolic compounds is really reduced. Regarding auxin content, we did not find a change in the expression of the auxin biosynthesis or conjugation genes, but it is possible that the transport can be altered due to the low expression of the *ABC4* transporter, which might be involved in auxin transport^46^, and the down-regulation of *WRKY23*. The Arabidopsis *WRKY23*, an auxin responsive gene^20^, has been also proposed to mediate auxin transport in the root^47^.

In Arabidopsis, proteins having a VQ motif, VQ proteins, interact with WRKY33. They act as activators of defense against necrotrophic pathogens^23^. Specifically, Arabidopsis VQ23 interacts with WRKY33 in SA- and JA-mediated defense response^23^. We found that silencing of *Fra a 1.02* caused down-regulation of *VQ25*, the corresponding gene in strawberry to Arabidopsis *VQ23*^48^. Moreover, the expression of this gene was enhanced in strawberry fruits after *Colletothricum* infection, and after MeJA and SA treatment of in vitro plants of this species^48^.

These results show that the defense response in strawberry fruits is altered in the *Fra a 1.02*-silenced lines, although the question remains as to whether this protein participates in the defense machinery. It is tempting to speculate that Fra a 1.02 participates early in the response to the pathogen, potentially after its interaction with a ligand, taking into account to the specific transcriptional changes triggered after its silencing in the ripe fruits, i.e., diminished expression of some regulatory *WRKY* genes and altered hormones content. All of these are associated with the response to pathogens in other species/organs. However, molecular interactions that support this putative role need to be studied in detail. To decipher it, it should be considered that Fra a 1 is a large family of proteins in strawberry that present a high level of sequence homology. Although, only a number of these proteins are present in the fruit, they show a very specific expression pattern that are different in the achene from the receptacle, and are variable during fruit development, ripening, and in different plant organs^4,5^. The crystal structure of Fra a 1.01 and Fra a 1.02, as well as an interacting protein partner of Fra a 1 proteins are known^11,12,49^, and this might help to reveal their molecular interactions. However, these two proteins have the capacity of interacting with natural ligands that trigger a conformational change in the proteins^11^, which might be critical for their roles in planta. A precise dissection of proteins, ligands, organs, developmental stages, and possible pathogens are needed for a significant advance in unraveling the molecular interactions that support a possible role of Fra a 1 proteins in plant defense.

## Materials and methods

### Plant transformation

All transformations were performed in *Fragaria x ananassa* Duch. cv. Camarosa, grown in a greenhouse under natural light and temperature ~24 °C, as previously reported^50^. The construct for the post-transcriptional silencing of *Fra a 1.02* was generated from a 124-bp fragment of the gene *FaFra a 1.02* (GenBank GQ148818) as shown in the Fig. S1. The nomenclature of *Fra a 1.01* to *Fra a 1.08* is based on the GenBank sequences for the *Fragaria x ananassa* genes^6^ and their corresponding genes in the sequenced genome of the diploid *Fragaria vesca*^7^ (Table S6). The fragment was cloned twice in antisense in the pHANNIBAL intermediate vector, and finally in a pBINPLUS vector. Three independent transgenic lines (L4, L5, and L6) were selected for analysis. Transformation with pBINPLUS empty vector were used as controls (pBIN4 and pBIN5). Transformations were checked in genomic DNA by PCR with specific primers for the insert and the kanamycin resistance gene.

### Protein analysis by western blot

Proteins were extracted from 100 mg of frozen tissue that was macerated at short intervals in a vortexer with liquid nitrogen and 100 μL of Laemmli 2X buffer. The extract was sonicated for 10 min at 4 °C. Then the extract was boiled for 5 min and centrifuged at 10,000 rpm for 8 min. The supernatant was stored at −20 °C until use. Equal amounts of total protein were loaded onto a 1-mm-thick 15% (w/v) polyacrylamide gel. Protein expression in *E. coli* and purification of recombinant proteins were performed as previously described^12^.

For western blotting, proteins were transferred from the SDS-PAGE gels to a Trans-Blot® nitrocellulose membrane using a Trans-Blot® Turbo™ Transfer System (Bio-Rad). After the transfer, the membrane was incubated with 5% blocking solution (5% fatty acid-free powder milk in TTBS buffer containing 20 mM Tris-HCl pH 7.6, 140 mM NaCl, and 0.05% v/v Tween20) for 2 h at room temperature, following an overnight incubation with the primary antibody at 4 °C. The anti-FaFra a 1.02 primary polyclonal antibody was used at a 1:500 dilution in 1% blocking solution (1% fatty acid free powder milk in TTBS buffer containing 20 mM Tris-HCl pH 7.6, 140 mM NaCl, 0.05% v/v Tween20). Chemiluminescent immunodetection was performed using EC, using either the normal Clarity™ Western ECL Substrate (BioRad) or the SuperSignal™ West Femto Maximum Sensitivity Substrate (Thermo Fisher) for low and high sensitivity, respectively. To generate the anti-FaFra a 1.02 polyclonal antibody, the purified full-length FaFra a 1.02 protein^11^ was used to immunize rabbits (Abyntek, Spain). Affinity purified fractions of the collected immune serum were used for western blot experiments. Anti-rabbit secondary antibodies were purchased from Santa Cruz Biotechnology (www.scbt.com).

### RNA extraction and expression analysis by RNAseq

Three pools for each line were used for RNA extraction, with at least five fruits from different plants of each line. Total RNA extraction was as previously reported^5^. RNA quality was evaluated with a Bioanalyzer 2100 (Agilent) and a NanoVue Plus (GE healthcare) using the A260/280 ratio. cDNA libraries of 200–500 bp fragments were prepared. Sequencing strategy included runs of 2X High Output V2, 150 cycles in the NextSeq550 (Ilumina), with 12 samples per run. The reads were mapped in the *F. vesca* genome^7^. Cleaning, counting, and annotation of the reads were performed with the programs TopHat, Cufflinks, and Cuffdiff^51^. Analysis of differentially expressed genes was performed with Cufflinks, establishing a *p*-value < 0.002 with the Benjamini–Hochberg procedure for multiple comparisons (http://cole-trapnelllab. github.io/cufflinks/cuffdiff/#differential-expression-tests). Functional assignment of the genes by gene ontology (GO) categories was done with Blast2go applied to the genes of *F. vesca*^7^.

### Analysis of metabolites

Samples of the fruits of the control and *Fra a 1.02*-silenced lines were individually frozen and homogenized with a mill to a fine powder. An aliquot of 500 mg of powder was used for each of the three biological replicates. The extraction and analysis of metabolites by liquid chromatography-electrospray ionization-mass spectrometry (LC-ESI-MSn) was performed as previously described^52^ with slight variations. A third extraction step with 500 µl methanol was added and the scan range was increased to a mass-to-charge ratio of 50–975. Metabolites were identified by their retention times, mass spectra, and product ion spectra in comparison with the data determined for authentic reference material. Biochanin A was used as internal standard (IS) for the relative quantification of the metabolites. Values are expressed in mg/kg-equivalent Biochanin A. Relative metabolite quantification was performed using the QuantAnalysis 6.2 software (Bruker Daltonics), normalizing all results to the IS. The software R (R: A Language and Environment for Statistical Computing, version 3.6.2) was used for statistical analysis of the metabolite contents. To find differences between the lines a Kruskal–Wallis test was performed followed by a Dunn-Bonferroni-Test using a *p*-value of ≤0.05 for significance.

### Analysis of hormones

Achenes were removed from frozen fruits and the receptacle was lyophilized for 48 h and ground to a fine powder. In total, 30 mg dry powder of the different repetitions of the samples were used for a double water extraction, in the presence of internal standards for ABA, SA, JA, and IAA, as previously described^53^. Basically, a pool of internal standards (IS) at 100 ng/ml containig abscisic acid‐d 6 (ABA‐d 6), salicylic acid‐d5 (SA‐d 5), indole acetic acid‐d 5 (IAA‐d 5), jasmonic acid-d6 (JA-d6), and N15-JA‐Ile‐d6 was added into each sample. Precise quantification was performed by using external calibration curves with each pure chemical, extraction yield was adjusted according to the recovery of the IS. After adjusting the pH to 2.5–2.7, extraction was carried out twice with diethyl ether, the organic fraction being dried in an evaporator at room temperature. The samples were resuspended in 1 ml of H_2_O/MeOH (90:10) with 0.01% of HCOOH. Chromatographic separation, identification of metabolites, data analysis and processing, was performed as previously reported^53^, with the only difference that the equipment used for IAA analysis was Xevo-TQS (Waters).

## Supplementary information

Supplementary figures

Table S1

Table S2

Table S3

Table S4

Table S5

Table S6

## References

[1] Fernandes H, Michalska K, Sikorski M, Jaskolski M (2013). Structural and functional aspects of PR-10 proteins. FEBS J..

[2] Liu JJ, Ekramoddoullah AKM (2006). The family 10 of plant pathogenesis-related proteins: their structure, regulation, and function in response to biotic and abiotic stresses. Physiol. Mol. Plant Pathol..

[3] Besbes F, Habegger R, Schwab W (2019). Induction of PR-10 genes an metabolites in strawberry plants in response to Verticillium dahliae infection. BMC Plant Biol..

[4] Ishibashi M (2018). Analysis of major paralogs encoding the Fra a 1 allergen based on their organ-specificity in Fragaria × ananassa. Plant Cell Rep..

[5] Sánchez-Sevilla JF (2017). Gene expression atlas of fruit ripening and transcriptome assembly from RNA-seq data in octoploid strawberry (Fragaria × ananassa). Sci. Rep..

[6] Franz-Oberdorf K (2016). Fra a 1.02 is the most potent isoform of the Betv1-like allergen in strawberry fruit. J. Agric. Food Chem..

[7] Edger PP (2018). Single-molecule sequencing and optical mapping yields an improved genome of woodland strawberry (Fragaria vesca) with chromosome-scale contiguity. GigaScience.

[8] Muñoz C (2010). The strawberry fruit fra a allergen functions in flavonoid biosynthesis. Mol. Plant.

[9] Kurze E, Kock V, Scalzo Lo R, Olbricht K, Schwab W (2018). Effect of the strawberry genotype, cultivation and processing on the Fra a 1 allergen content. Nutrients.

[10] Hjernø K (2006). Down‐regulation of the strawberry Bet v 1‐homologous allergen in concert with the flavonoid biosynthesis pathway in colorless strawberry mutant. Proteomics.

[11] Casañal A (2013). The strawberry pathogenesis-related 10 (PR-10) Fra a proteins control flavonoid biosynthesis by binding to metabolic intermediates. J. Biol. Chem..

[12] Orozco-Navarrete, B. et al. Structural bases for the allergenicity of Fra a 1.02 in strawberry fruits. *J. Agric. Food Chem.* **68**, 10951–10961 (2020). 10.1021/acs.jafc.9b05714 PMC7644122 31774998

[13] Seutter von Loetzen C (2014). Secret of the major birch pollen allergen Bet v 1: identification of the physiological ligand. Biochem. J..

[14] Noctor G (2012). Glutathione in plants: an integrated overview. Plant Cell Environ..

[15] Heeg C (2008). Analysis of the Arabidopsis O-acetylserine(thiol)lyase gene family demonstrates compartment-specific differences in the regulation of cysteine synthesis. Plant Cell.

[16] Watanabe M, Tohge T, Fernie AR, Hoefgen R (2018). The effect of single and multiple SERAT mutants on serine and sulfur metabolism. Front. Plant Sci..

[17] Dixon DP, Lapthorn A, Edwards R (2002). Plant glutathione transferases. Genome Biol..

[18] Sylvestre-Gonon E (2019). Functional, structural and biochemical features of plant serinyl-glutathione transferases. Front. Plant Sci..

[19] Upadhyay N (2019). The multitasking abilities of MATE transporters in plants. J. Exp. Bot..

[20] Prát T (2018). WRKY23 is a component of the transcriptional network mediating auxin feedback on PIN polarity. PLoS Genet..

[21] Timmermann T (2019). Gene networks underlying the early regulation of Paraburkholderia phytofirmans PsJN induced systemic resistance in Arabidopsis. PLoS ONE.

[22] Zheng Z, Qamar SA, Chen Z, Mengiste T (2006). Arabidopsis WRKY33 transcription factor is required for resistance to necrotrophic fungal pathogens. Plant J..

[23] Cheng Y (2012). Structural and functional analysis of VQ motif-containing proteins in Arabidopsis as interacting proteins of WRKY transcription factors. Plant Physiol..

[24] Amil-Ruiz F (2016). Partial activation of SA- and JA-defensive pathways in strawberry upon colletotrichum acutatum interaction. Front. Plant Sci..

[25] Wasternack C, Song S (2017). Jasmonates: biosynthesis, metabolism, and signaling by proteins activating and repressing transcription. J. Exp. Bot..

[26] Chini A, Gimenez-Ibanez S, Goossens A, Solano R (2016). Redundancy and specificity in jasmonate signalling. Curr. Opin. Plant Biol..

[27] Dempsey DA, Vlot AC, Wildermuth MC, Klessig DF (2011). Salicylic acid biosynthesis and metabolism. Arabidopsis Book.

[28] Yang Y (2008). Inactive methyl indole-3-acetic acid ester can be hydrolyzed and activated by several esterases belonging to the AtMES esterase family of Arabidopsis. Plant Physiol..

[29] Fu J, Wang S (2011). Insights into auxin signaling in plant-pathogen interactions. Front. Plant Sci..

[30] Kaiser R (2016). Genotyping of red and white fruited strawberry (Fragaria L.) accessions and hybrids based on microsatellite markers and on the genetic diversity in the allergen genes *fra a 1* and *fra a 3*. Genet. Resour. Crop Evol.

[31] Härtl K (2017). Early metabolic and transcriptional variations in fruit of natural white-fruited Fragaria vesca genotypes. Sci. Rep..

[32] Francisco RM (2013). ABCC1, an ATP binding cassette protein from grape berry, transports anthocyanidin 3-O-Glucosides. Plant Cell..

[33] Gomez C (2009). Grapevine MATE-type proteins act as vacuolar H+-dependent acylated anthocyanin transporters. Plant Physiol..

[34] Sun Y, Li H, Huang JR (2012). Arabidopsis TT19 functions as a carrier to transport anthocyanin from the cytosol to tonoplasts. Mol. Plant.

[35] Luo H (2018). Reduced Anthocyanins in Petioles codes for a GST anthocyanin transporter that is essential for the foliage and fruit coloration in strawberry. J. Exp. Bot..

[36] Takanashi K, Shitan N, Yazaki K (2014). The multidrug and toxic compound extrusion (MATE) family in plants. Plant Biotechnol..

[37] Chen SY (2018). FaTT12-1, a multidrug and toxin extrusion (MATE) member involved in proanthocyanidin transport in strawberry fruits. Sci. Hortic..

[38] Ishihara T (2008). Overexpression of the Arabidopsis thaliana EDS5 gene enhances resistance to viruses. Plant Biol..

[39] Sun X (2011). ADS1 encodes a MATE-transporter that negatively regulates plant disease resistance. New Phytol..

[40] Chen F (2017). The WRKY transcription factor family in model plants and crops. Crit. Rev. Plant Sci..

[41] Wei W (2018). Ectopic expression of FvWRKY42, a WRKY transcription factor from the diploid woodland strawberry (Fragaria vesca), enhances resistance to powdery mildew, improves osmotic stress resistance, and increases abscisic acid sensitivity in Arabidopsis. Plant Sci..

[42] Caarls L, Pieterse CMJ, Van Wees SCM (2015). How salicylic acid takes transcriptional control over jasmonic acid signaling. Front. Plant Sci..

[43] Higuera JJ (2019). The strawberry FaWRKY1 transcription factor negatively regulates resistance to colletotrichum acutatum in fruit upon infection. Front. Plant Sci..

[44] Robert-Seilaniantz A, Grant M, Jones JDG (2011). Hormone crosstalk in plant disease and defense: more than just JASMONATESALICYLATE antagonism. Annu. Rev. Phytopathol..

[45] Gamir J (2018). Starch degradation, abscisic acid and vesicular trafficking are important elements in callose priming by indole‐3‐carboxylic acid in response to Plectosphaerella cucumerina infection. Plant J..

[46] Cho M, Lee ZW, Cho HT (2012). ATP-binding cassette B4, an auxin-efflux transporter, stably associates with the plasma membrane and shows distinctive intracellular trafficking from that of PIN-FORMED proteins. Plant Physiol..

[47] Grunewald W (2012). Transcription factor WRKY23 assists auxin distribution patterns during Arabidopsis root development through local control on flavonol biosynthesis. Proc. Natl Acad. Sci. USA.

[48] Garrido-Gala J, Higuera JJ, Muñoz-Blanco J, Amil-Ruiz F, Caballero JL (2019). The VQ motif-containing proteins in the diploid and octoploid strawberry. Sci. Rep..

[49] Franz-Oberdorf K (2017). Physical interaction between the strawberry allergen Fra a 1 and an associated partner FaAP: interaction of Fra a 1 proteins and FaAP. Proteins.

[50] Vallarino JG (2020). Characterizing the involvement of FaMADS9 in the regulation of strawberry fruit receptacle development. Plant Biotechnol. J..

[51] Trapnell C (2012). Differential gene and transcript expression analysis of RNA-seq experiments with TopHat and Cufflinks. Nat. Protoc..

[52] Ring L (2013). Metabolic interaction between anthocyanin and lignin biosynthesis is associated with peroxidase FaPRX27 in strawberry fruit. Plant Physiol..

[53] Sánchez-Bel P (2018). Mycorrhizal tomato plants fine tunes the growth-defence balance upon N depleted root environments. Plant Cell Environ..

