# Supplementary material for: Down-regulation of Fra a 1.02 in strawberry fruits causes transcriptomic and metabolic changes compatible with an altered defense response
Source: Hortic Res. 2021 Mar 10;8:58. doi: 10.1038/s41438-021-00492-4 (PMC7943815; doi:10.1038/s41438-021-00492-4)
Supplement: Supplementary file 1 — Supplementary figures [file 41438_2021_492_MOESM1_ESM.pdf]

Figure S1

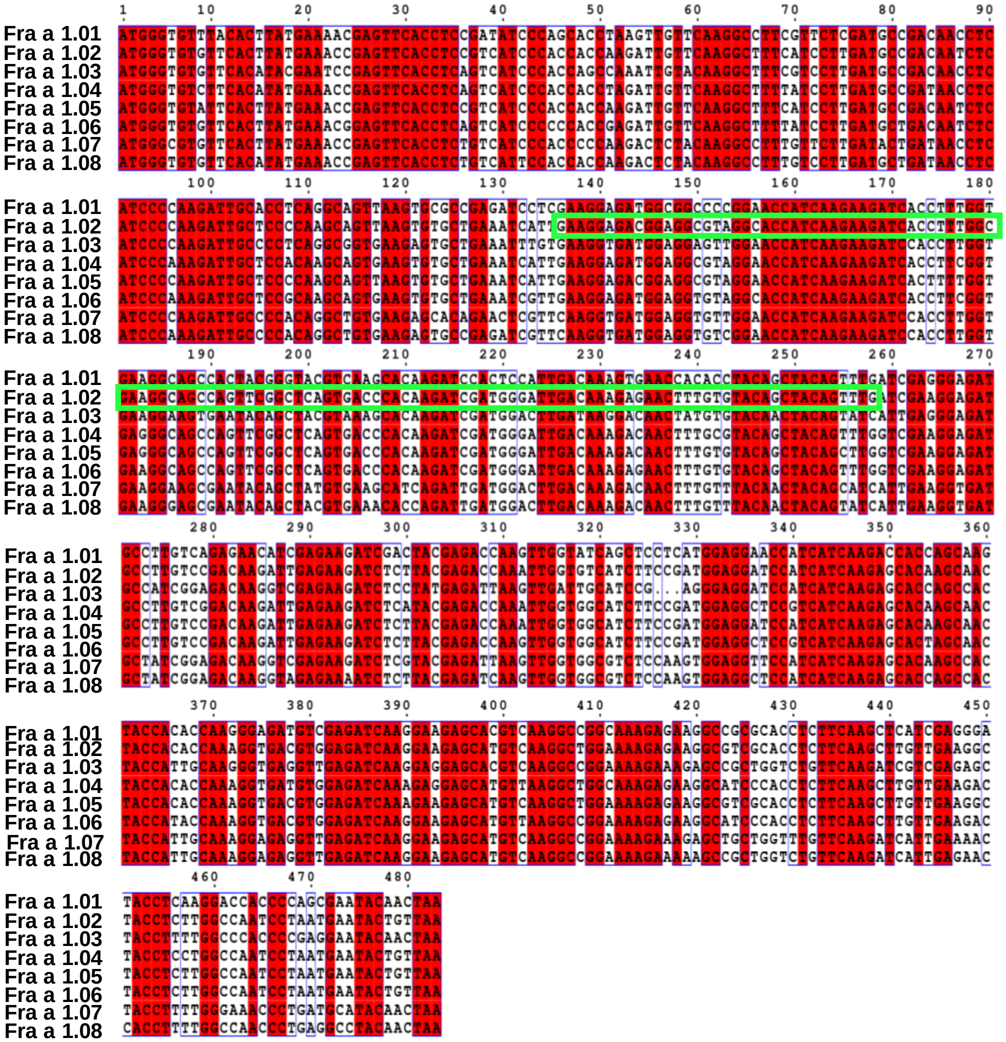

Fra a 1.01
Fra a 1.02
Fra a 1.03
Fra a 1.04
Fra a 1.05
Fra a 1.06
Fra a 1.07
Fra a 1.08

Figure S2

Gen	35S::Fra a 1.02i (L4)	35S::Fra a 1.02i (L5)	35S::Fra a 1.02i (L6)	Mean
Fra a 1.01	51,7	46,0	57,7	**51,8**
Fra a 1.02	96,6	85,3	94,6	**92,2**
Fra a 1.03	66,9	27,1	64,3	**52,7**
Fra a 1.04	97,3	83,4	98,4	**93,1**
Fra a 1.05	95,0	88,7	96,8	**93,5**
Fra a 1.06	97,8	90,3	97,0	**95,1**
Fra a 1.07	78,1	28,4	67,7	**58,2**
Fra a 1.08	16,3	29,7	20,2	**22,1**

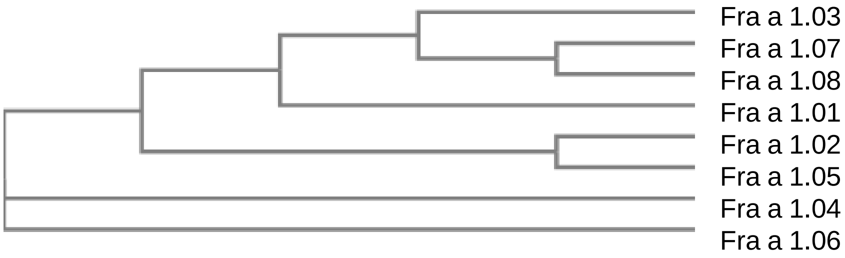

Figure S3

Biological Process

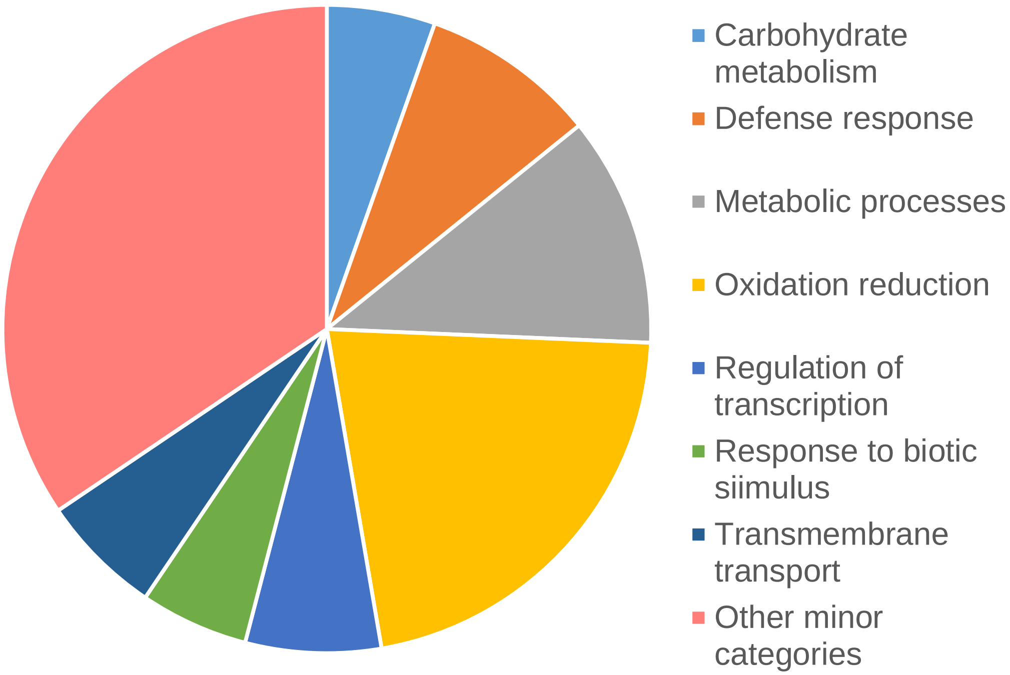

Cell component

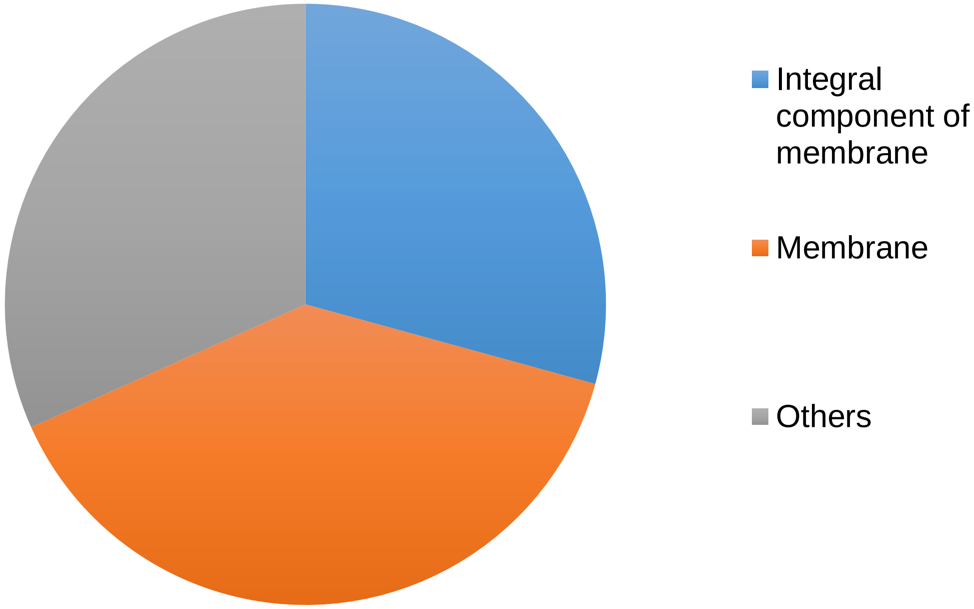

Figure S4

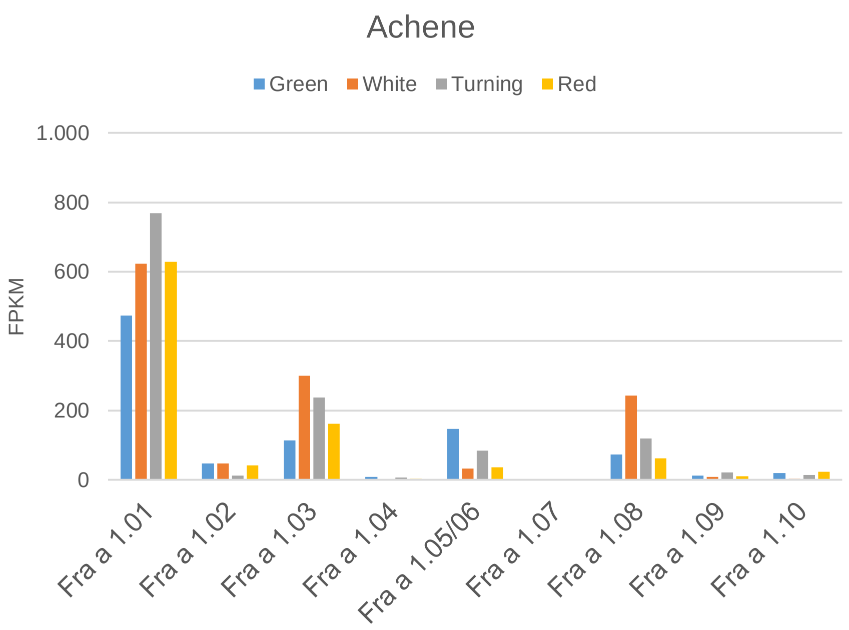
Achene
Green
White
Turning
Red
FPKM
1.000
800
600
400
200
0
Fra a 1.01
Fra a 1.02
Fra a 1.03
Fra a 1.04
Fra a 1.05/06
Fra a 1.07
Fra a 1.08
Fra a 1.09
Fra a 1.10

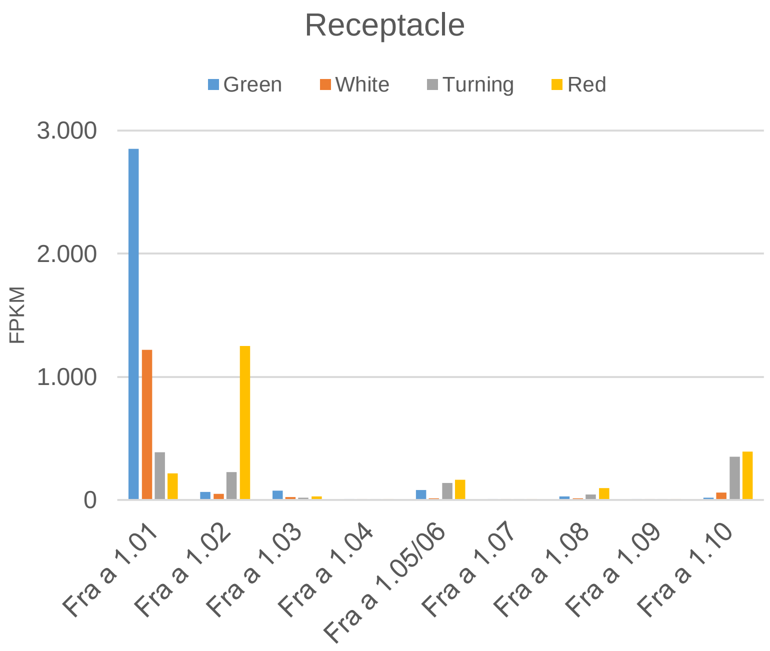
Receptacle
Green
White
Turning
Red
FPKM
3.000
2.000
1.000
0
Fra a 1.01
Fra a 1.02
Fra a 1.03
Fra a 1.04
Fra a 1.05/06
Fra a 1.07
Fra a 1.08
Fra a 1.09
Fra a 1.10
